# Functional Cross Talk between CXCR4 and PDGFR on Glioblastoma Cells Is Essential for Migration

**DOI:** 10.1371/journal.pone.0073426

**Published:** 2013-09-02

**Authors:** Miriam Sciaccaluga, Giuseppina D’Alessandro, Francesca Pagani, Giuseppina Ferrara, Nadia Lopez, Tracy Warr, Paolo Gorello, Alessandra Porzia, Fabrizio Mainiero, Antonio Santoro, Vincenzo Esposito, Giampaolo Cantore, Emilia Castigli, Cristina Limatola

**Affiliations:** 1 IRCCS Neuromed, Venafro, Italy; 2 Istituto Pasteur Fondazione Cenci Bolognetti, Dipartimento di Fisiologia e Farmacologia Sapienza University of Rome, Rome, Italy; 3 Centre for Life Nano Science@Sapienza, Istituto Italiano di Tecnologia, Rome, Italy; 4 Department of Cellular and Environmental Biology, University of Perugia, Perugia, Italy; 5 Hematology, University of Perugia, Perugia, Italy; 6 Department of Molecular Medicine, Sapienza University of Rome, Rome, Italy; 7 Department of Experimental Medicine, Sapienza University of Rome, Rome, Italy; 8 Department of Neurology and Psychiatry, Sapienza University of Rome, Rome, Italy; The University of Chicago, United States of America

## Abstract

Glioblastoma (GBM) is the most common and aggressive form of brain tumor, characterized by high migratory behavior and infiltration in brain parenchyma which render classic therapeutic approach ineffective. The migratory behaviour of GBM cells could be conditioned by a number of tissue- and glioma-derived cytokines and growth factors. Although the pro-migratory action of CXCL12 on GBM cells in vitro and in vivo is recognized, the molecular mechanisms involved are not clearly identified. In fact the signaling pathways involved in the pro-migratory action of CXCL12 may differ in individual glioblastoma and integrate with those resulting from abnormal expression and activation of growth factor receptors. In this study we investigated whether some of the receptor tyrosine kinases commonly expressed in GBM cells could cooperate with CXCL12/CXCR4 in their migratory behavior. Our results show a functional cross-talk between CXCR4 and PDGFR which appears to be essential for GBM chemotaxis.

## Introduction

Glioblastoma (GBM) is the most aggressive form of human brain tumors, its poor prognosis largely deriving from the high invasiveness throughout the brain parenchyma, which is the leading cause of the resistance to traditional therapeutic approaches [1,2]. Invasion thus appears to be a key target in contrasting this kind of tumor and, in recent years, a number of studies have been directed at understanding the molecular mechanisms underlying GBM cell migration and invasion and the complex network of interactions achieved with the surrounding brain tissue, which contribute to promoting the motility and maintaining the path of invasion.

Growth factors, cytokines, chemokines and their receptors are key players of these multifactorial signaling systems arising in various districts within the tumor mass as result of interactions with the infiltrated normal tissue [3–6]. The cross-talk between cell-surface receptors and the redundancy of downstream effectors makes the individuation of invasion leading signals even more complex.

A large body of information points to crucial role of the chemokine CXCL12 and its receptor CXCR4 in the migratory behavior of GBM cells, both *in vivo* and *in vitro* [7,8]. Several lines of evidence led to the concept that the CXCL12/CXCR4 axis is a key effector of the nonrandom typical invasive pattern of human GBM [9]: the overexpression of CXCR4 in the invasive GBM cells [4]; the *in vivo* localization of CXCR4 in the hypoxic areas [10], considered the basis for the acquisition of a highly invasive phenotype [11]; the demonstration that CXCR4 expression is under the control of HIF1 and VEGF [12].

The migratory behavior of GBM cells may be conditioned by the action of growth factors and their receptors, which are often over-expressed or constitutively active in GBM cells. Several studies demonstrated the existence of different combinations of abnormal expression and activation of growth factor receptors (such as EGFR, PDGFRα, PDGFRβ, c-kit, met, and ret) in GBM-derived cell lines and primary cultures, suggesting that the co-activation of these receptors may condition the response of GBM cells to targeted therapies [13].

Among the growth factors potentially involved in the migratory capability of GBM, the most studied is the EGF, since its receptor has been demonstrated to be over-expressed or mutated in a large percentage (40%) of glioblastomas [14]. The altered expression of EGFR in human GBM is generally correlated with high proliferative behavior and with resistance to apoptosis although its involvement in the acquisition of the migratory phenotype could be inferred by the demonstration that EGFR over-expression confers migratory properties to otherwise non-migrating neural progenitor cells [15] and that EGF can act as a potent motogen for GBM cells [6]. It is interesting to note that the abnormal expression of EGFR has been demonstrated to be associated with the activation of CXCR4 in GBM biopsies, and that EGF is able to induce CXCR4 phosphorylation in EGFR over-expressing GBM cells [16]. This kind of finding highlights the possibility of a cross-talk between CXCR4 and abnormally activated RTKs in GBM cells.

Platelet-derived growth factors (PDGFs) and their receptors, up-regulated in at least a third of surgical glioma samples and human glioma cell lines, have been extensively demonstrated to be involved in proliferation, cell migration, and angiogenesis of GBM cells [17]. Their involvement in gliomagenesis is further strengthened by a recent definition of GBM subclasses, where the PDGF class was characterized by high levels of PDGFBB ligand and phosphorylation of PDGFRβ [18]. A possible cross-talk between CXCL12/CXCR4 axis and PDGFRs is highlighted by the demonstration that the response to STI571, an inhibitor of PDGFR family members, is conditioned by CXCL12 expression in GBM cells [19]. The cross-talk between GPCRs and RTKs is not a new concept, because in the last decade a large body of information indicates that GPCRs and RTKs, that activate a common set of signaling molecules, do not operate in an isolated fashion [20–23]. Moreover, in GBM cells, the over-expression and/or increased activity of RTKs could strength the crosstalk with GPCRs, highlighting the possibility of specific therapeutic strategies targeting signaling molecules activated by the interaction between RTKs and GPCRs. Therefore we hypothesized that the abnormal activities of RTKs, in particular PDGFRβ, in GBM cells could cooperate with CXCL12/CXCR4 axis in modulating their migratory behavior.

In this paper we demonstrate that PDGFRβ and CXCR4 are co-expressed in GBM cells and that these receptors functionally interact to modulate cell migration.

## Materials and Methods

### Materials

Cell culture medium (Dulbecco’s modified minimum essential medium, DMEM), fetal bovine serum (FBS), penicillin G, streptomycin, glutamine, and sodium pyruvate were from GIBCO Invitrogen (Carlsbad, CA); recombinant human CXCL12 was from Peprotech (London, UK); and recombinant human PDGFBB and Fura-2 AM were from Invitrogen (Carlsbad, CA). Antibody anti-CXCR4 and anti phospho-CXCR4 (Ser339) were from Abcam (Cambridge, UK), anti-phospho PDGFRβ (Tyr751), anti PDGFRβ, anti PDGFRα, anti c-kit anti-phospho-p44/42 MAPK (Thr202/Tyr204) E10, anti-p44/42 MAPK, and ECL were from Cell Signaling Technology (Beverly, MA); anti-CXCR4 APC and anti-PDGFRβ PE were from BD Biosciences (Buccinasco, Italy); AG1296 (IC_50_ for PDGFRβ, 800 nM), PDGFR inhibitor VII (IC_50_, 10 nM), PP2 (IC_50_ for src, 100 nM), AG1478 (IC_50_ for EGFR, 3 nM), and BAPTA-AM were from Calbiochem (San Diego, CA); AMD-3100 (IC_50_ for CXCR4, 20-130 nM), DMSO, secondary antibodies, and all the other chemicals were from Sigma-Aldrich (St. Louis, MO) or Pierce (Rockford, IL).

### Cell cultures

The human GBM cell line GL15 was grown in DMEM supplemented with 10% heat-inactivated FBS, 100 IU/ml penicillin G, 100 µg/ml streptomycin, 2 mM glutamine, and 1 mM sodium pyruvate. Cells were grown at 37°C in a 5% CO_2_ humidified atmosphere and always used between passages 40-60 [24]. Medium was changed twice a week and the cells were sub-cultured when confluent. In some experiments, cells were maintained in serum-free medium for 16-18 h before experiments.

### Freshly dissociated GBM cells from patients

Tumor *specimens* were obtained from the Department of Neurology and Psychiatry of Sapienza Medical School and from Department of Neurosurgery, Neuromed, from GBM patients who gave a written informed consent to the research proposals. The study was approved by the Institutional Ethics Committee of Neuromed and Sapienza University and by Ministry of Health. Tissues were processed within half an hour from surgical resection. Histopathological typing and tumor grading were done according to the WHO criteria resulting as grade IV. In detail, tumor tissues were mechanically dissociated to cell suspensions and centrifuged at 800 g for 5 min. Residual red blood cells were lysed with 4 vol of ammonium chloride buffer (in mM: 154 NH_4_Cl, 10 KCO_3_, and 0.1 EDTA) at 4°C for 5 min. Tumor cells were re-suspended in serum-free growth medium (DMEM with 100 IU/ml penicillin G, 100 µg/ml streptomycin, 4 mM glutamine, and 1 mM sodium pyruvate) and cultured at 37°C in humidified atmosphere with 5% CO_2_. Twenty-four h later, nonadherent cells were removed and the growth medium was supplemented with 10% heat-inactivated FBS, with changes every 48 h. After about 10 days, cells were sub cultured only once and then used for immunocytochemical characterization (see 25) and chemotaxis experiments. These freshly dissociated cells are named GBM9, GBM10, GBM12, GBM13, GBM18, GBM19, GBM21, GBM46, GBM47.

### Immunoblotting

GL15 cells at one day of subculture were serum starved for 30 min in the presence or absence of AG1296 (20 µM) and then stimulated with recombinant human CXCL12 (50 nM) or with recombinant human PDGFBB (50 ng/ml) for 15 min. Cell cultures were washed with PBS and scraped with 62.5 mM Tris·HCl (pH 6.8), 2 mM EDTA, 0.5% Triton X-100, phosphatase and protease inhibitor cocktails and 2% SDS. The proteins were separated by SDS-PAGE and transferred to nitrocellulose filters. Immunolabeling was performed following the instructions of the manufacturer. Enhanced chemiluminescence detection was performed and band intensity quantified by ChemiDoc (BioRad).

### Cytofluorimetric analysis

GL15 cells were washed with PBS and stained with anti-CXCR4 APC or anti-PDGFRβ PE or with a combination of both mAbs for 30 min at 4°C. Cells were analyzed with a FACScalibur (BD Biosciences), using FlowJo (Treestar, Ashland, OR) software. GL15 population was defined on the basis of forward and side scatter physical parameters (data not shown); the profiles of CXCR4- or PDGFRβ-expressing GL15 cells and of PDGFRβ-expressing CXCR4-high- and CXCR4-low GL15 cells were obtained compared to matched isotype controls (APC or PE IgG2a stained).

### Intracellular calcium measurements

Fluorescence determinations were performed by a conventional fluorescence microscopy system composed of an upright microscope (Axioskop; Zeiss, Jena, Germany), a digital 12-bit cooled camera (SensiCam) and a monochromator (Till Photonics). The system was driven by Till Vision software (Till Photonics). Images were acquired and stored on a Dell PC, then analyzed offline. Measurements of fluorescence over time had a resolution of 0.5 Hz. GL15 cells were plated at 100.000/ml and used after 4 days of culture. Twenty-four h before the experiment, cultures were wounded with a plastic tip, to create an empty space that allows visualization of migrating cells. Before the experiments, cells were incubated with Fura-2 AM (3 µM) for 45 min and then extensively washed with external solution of the following composition (in mM): 140 NaCl, 5.6 KCl, 2 MgCl_2_, 2 CaCl_2_, 10 glucose, and 10 HEPES-NaOH, pH 7.4. Imaging fields were chosen in the wound area. Emission was monitored at 510 nm (optical filters and dichroic beam splitter were from Chroma, Brattleboro, VT). Drugs were applied by a gravity-driven perfusion system, with tubing connected to a final tip of 100-200 µm diameter, focally oriented onto the field of interest.

### Chemotaxis assay

CXCL12- or PDGFBB-induced chemotaxis was investigated in GBM cell lines, primary cultures and HEK cells. Semiconfluent cells were trypsinized, preincubated in chemotaxis medium (DMEM without glutamine, 100 IU/ml penicillin G, 100 µg/ml streptomycin, 0.01% BSA, and 25 mM HEPES, pH 7.4) for 15 min, and plated (500.000/well) on 10.5-mm polylysine-coated transwells (8 µm pore size filters) in this same medium. When necessary, cells were preincubated 15 min with AG1296 (20 µM), AMD-3100 (1 µg/ml), AG1478 (250 nM), which were also present during the assay. In all the experiments the final concentration of DMSO was 0.1%. The lower chambers contained chemotaxis medium enriched with CXCL12 (50 nM), PDGFBB (50 ng/ml), or vehicle. After 4 h of incubation at 37°C, cells were treated with ice-cold 10% trichloroacetic acid for 10 min. Cells adhering to the upper side of the filter were scraped off, whereas cells on the lower side were stained with a solution containing 50% isopropanol, 1% formic acid, and 0.5% (w/vol) brilliant blue R 250. Stained cells were counted in more than 20 fields with a ×40 objective.

### RT-PCR

One µg of total RNA was reverse transcribed using Thermoscript RT-PCR system (Invitrogen). The following primers were used for amplification: *PDGFRα F*, 5’-CAAAGTGGAGGAGACCATCG-3’; *PDGFRα R*, 5’-TGAGAGCTTGTTTTTCACTGGA -3’; *PDGFRβ F*, 5’-CACTGCCTGTCCCCTATGAT-3’; *PDGFRβ R*, 5’-TTGACGGCCACTTTCATCGT -3’; KIT F, 5’- CCACACCCTGTTCACTCCTT -3’; KIT R, 5’-TGCATGATCTTCCTGCTTTG-3’. DNA was denatured at 94 °C for 4 min, then subjected to 35 cycles with denaturing at 94 °C for 30 sec annealing at 57.5 °C (PDGFRα) or 58°C (PDGFRβ and KIT) for 30 sec and elongation at 72 °C for 60 sec, followed by final elongation at 72 °C for 5 min. Ampli Taq Gold DNA polymerase (Roche) was used for all PCR reactions.

## Results

### CXCR4 and PDGFR cross-talk is essential for chemotaxis of GL15 cells

The migratory behaviour of GBM cells is known to be conditioned by a number of tissue- and glioma-derived cytokines and growth factors [26] and among these, CXCL12 modulates different aspects of glioma biology, including migration [7,8,25].

**Figure 1:**
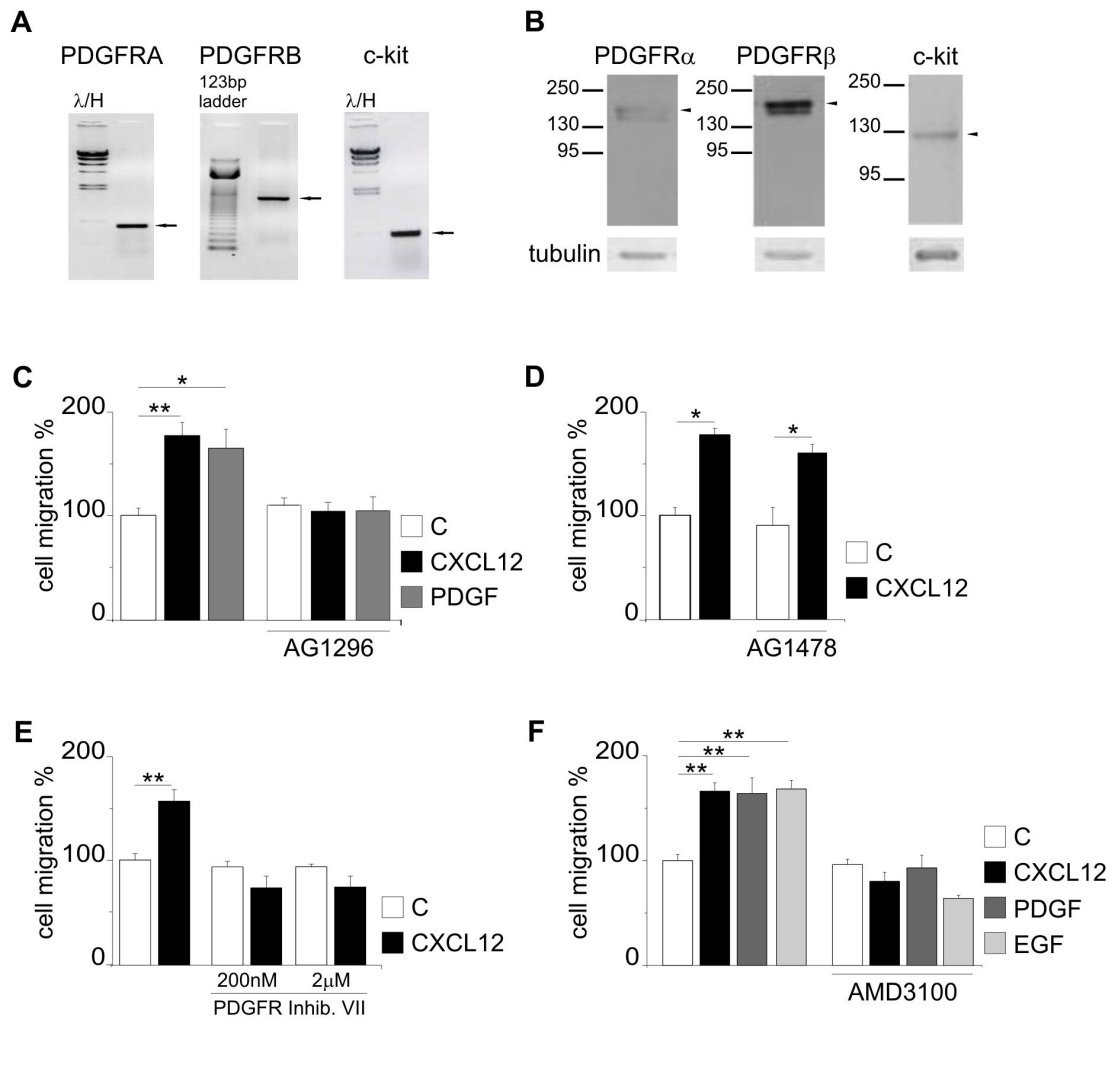
GL15 cells express functional PDGFR and its activity is necessary for chemotactic response to CXCL12. The expression of PDGFR family members is evaluated by RT-PCR (A) and Western Blotting (B) in GL15 cells. C) Effect of AG1296 (20 µM) on CXCL12- and PDGFBB-induced chemotaxis. D) Effect of AG1478 (250 nM) on CXCL12-induced chemotaxis. E) Effect of PDGFR inhibitor VII on CXCL12-induced chemotaxis. F) Effect of AMD3100 (1 µg/ml) on CXCL12- and PDGFBB-induced chemotaxis. In C, D, E and F, results are reported as percentage of cell migration in stimulated *vs* un-stimulated samples in the presence or in the absence of the indicated inhibitors (all pre-incubated for 15 min) and are the mean ± SE of at least three independent experiments. Statistical significance: *P<0.05, **P<0.01; one way ANOVA.

Since EGFR and PDGFR family members have been demonstrated to be abnormally expressed or activated in a large percentage of GBM, we hypothesized a possible involvement of these receptors in CXCL12-induced GBM cell migration. As a first step to test this hypothesis we analyzed the expression of PDGFR family members in the human GBM cell line GL15, which has been demonstrated to be tumorigenic and highly invasive in *in vivo* experimental models [27], and to over-express EGFR [28]. RT-PCR and western blot analyses showed that GL15 cells express PDGFRα, PDGFRβ and c-kit (Figure 1 A,B). To investigate the involvement of PDGFR activation in GL15 cells migration, transwell chemotaxis assays were performed in the presence of PDGFBB, able to activate both PDGFRα and β. Results obtained demonstrated that these cells migrate toward PDGF (Figure 1C), with a chemotactic index similar to that obtained with CXCL12. PDGF-induced GL15 cell migration was efficiently blocked by AG1296 (20 µM), a specific inhibitor of PDGFR family members (Figure 1C). Interestingly, AG1296 also efficiently inhibited CXCL12-induced cell chemotaxis (Figure 1C) while AG1478 (250 nM), a selective inhibitor of EGFR activation, did not affect CXCL12-induced migration (Figure 1D), thus suggesting a possible involvement of PDGFR activity on CXCL12-induced chemotaxis. To exclude non-specific effects of AG1296 on CXCR4, chemotaxis experiments were performed on HEK cells. On these cells PDGFBB and CXCL12 did not induce cell migration; upon CXCR4 transfection, CXCL12-induced cell chemotaxis and this activity was not inhibited by AG1296 treatment (Figure S1 A and B).

To further confirm the involvement of PDGFR activity on CXCL12-induced GL15 migration, chemotaxis assays were performed in the presence of the PDGFR tyrosine kinase inhibitor VII, a selective inhibitor of PDGFRβ [29]. Our results show that inhibitor VII is able to block CXCL12-induced chemotaxis, thus confirming the role of PDGFRβ activity in this phenomenon (Figure 1E). When GL15 cells were treated with AMD3100, a CXCR4 inhibitor, we observed a completely counteraction of PDGF-induced chemotaxis (Figure 1F), highlighting a bidirectional crosstalk between PDGFR and CXCR4. Interestingly, AMD3100 also abolished EGF induced cell migration, suggesting that also in GL-15 GBM there is a functional interaction between these two receptors [30]. In control experiments, AMD3100 abolished CXCL12-induced GL15 cell migration (Figure 1F). To exclude non specific effects of AMD3100 on PDGFR, chemotaxis experiments were performed on two primary GBM cells obtained from patients that were not responsive to CXCL12 but expressed PDGFRβ, GBM12 and GBM13 (see below, Tables 1 and 2). Data obtained indicate that in these cells PDGF induced migration and that AMD3100 did not affect cell migration (Figure S 1 C and D).

**Table 1:** Expression of CXCR4, PDGFRα, PDGFRβ and c-kit in primary cultures of human GBM biopsies

***Cellculture***	**CXCR4**	**PDGFRα**	**PDGFRβ**	**c-kit**
GBM9	++	+++	+++	++
GBM10	ND	ND	+	++
GBM12	ND	ND	++	-
GBM13	+	+++	++	+
GBM18	++	-	-	+
GBM19	++	+++	+	-
GBM21	++	-	-	++
GBM46	+	++	+++	+
GBM47	++	+	+	+

Cells were divided in 4 groups according to the expression of each receptor relative to tubulin (as ratio of densitometric analyses): - no expression (0); + weak expression (1–60); ++ high expression (61–150); +++ very high expression (more than 150). ND: not determined.

**Table 2:** Effects of AG1296 on CXCL12-induced chemotaxis in primary cultures of human GBM biopsies.

***cellculture***	**C**	**CXCL12**	**AG1296**	**AG1296+CXCL12**
GBM9	**100 ± 8.1**	200 ± 18.8**	100 ± 8.5	91 ± 18.8
GBM10	**100 ± 8.9**	154 ± 11.5*	100 ± 7.6	87 ± 6.3
GBM12	100 ± 4.1	108 ± 12.2	100 ± 29.6	113 ± 26.2
GBM13	100 ± 1.1	114 ± 8	100 ± 7.5	94 ± 3.7
GBM18	**100 ± 9.7**	152 ± 15.6*	100 ± 4	116 ± 14.3
GBM19	**100 ± 2.8**	176 ± 10.8**	100 ± 7.8	92 ± 7.1
GBM21	**100 ± 4.9**	160 ± 23.3*	100 ± 12.4	109 ± 10.2
GBM46	**100 ± 11.1**	160 ± 16.8*	100 ± 12.6	90 ± 17.7
GBM47	100 ± 36	148 ± 44.3	100 ± 14.6	105 ± 22.4

Results are reported as percentage of cell migration in stimulated *vs* un-stimulated samples in the presence or in the absence of AG1296 (pre-incubated for 15 min) and are the mean ± SE of at least three independent experiments. Statistical significance: *P<0.05, **P<0.01, Student’s *t* test.

When experiments were conducted in the absence of serum to better mimic the conditions of GBM growth *in vivo*, the same results were obtained with CXCL12, PDGF and the two receptor inhibitors (Figure S2).

### CXCR4 and PDGFR are co-expressed in GL15 cells

**Figure 2:**
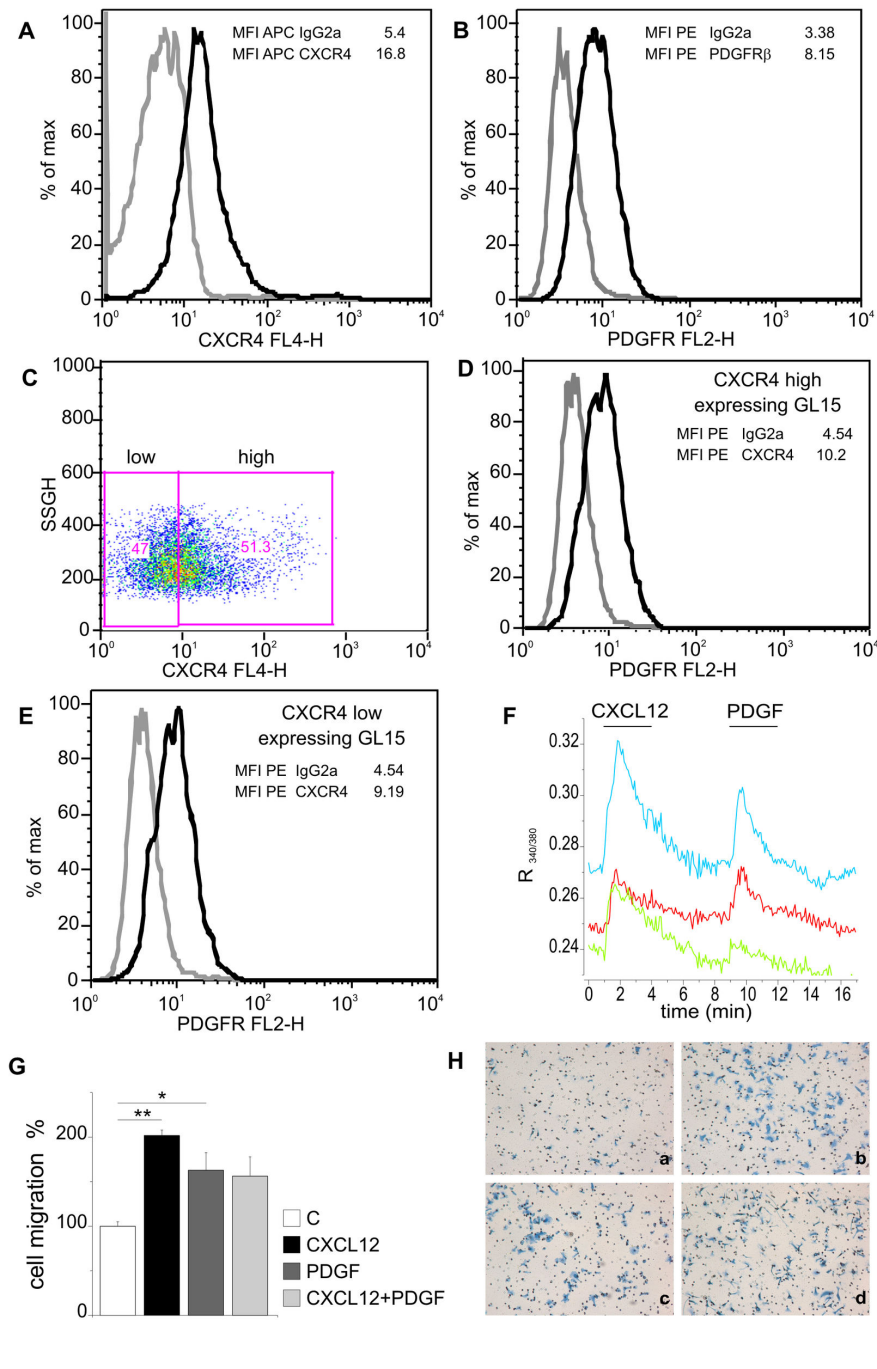
CXCR4 and PDGFR are co-expressed in GL15 cells. FACS analysis representing the profiles of A) CXCR4 (black line) expressing GL15 cells compared to matched isotype APC IgG2a control (grey line); B) PDGFRβ (black line) expressing GL15 cells compared to matched isotype PE IgG2a control (grey line); C) Dot Plot of CXCR4 high and CXCR4 low expressing GL15 cells; D) PDGFRβ expression in CXCR4 high expressing GL15 cells (black line) compared to matched isotype PE IgG2a control (grey line); E) PDGFRβ expression in CXCR4 low expressing GL15 cells (black line) compared to matched isotype PE IgG2a control (grey line). The mean fluorescence intensity (MFI) of CXCR4 or PDGFRβ stained samples (black lines) and APC or PE IgG2a stained controls (grey lines) are shown in panels A-E. F) Fluorescence transients in Fura2-loaded GL-15 cells superfused with CXCL12 (50 nM; 3 min) and, after 5 min, with PDGFBB (50 ng/ml; 3 min) as indicated. Traces, expressed as changes in 340/380 nm fluorescence ratio (R), represent the responses from 3 representative cells responding to both agonists within the same field. G) Chemotaxis assay towards CXCL12, PDGF or CXCL12/PDGF show no synergistic increase in cell migration. Data in G as in Figure 1, are the mean ± S.E. of at least three independent experiments. Statistical significance: *P<0.05, **P<0.01, one way ANOVA. H) Representative filters of chemotaxis experiment showing GL15 cells migrating in control (a), and toward CXCL12 (b), PDGF (c) or CXCL12 + PDGF (d).

To investigate the possibility of an intracellular cross-talk between CXCR4 and PDGFR, we first analyzed whether CXCR4 and PDGFR were present on the same subsets of GL15 cells. For these experiments, GL15 cells were incubated with antibodies against CXCR4 and PDGFR and analyzed by FACS. Results, shown in Figure 2 A,B demonstrate that all GL15 cells were positively stained for both receptors. Figure 2 C illustrates that CXCR4 is expressed at different levels (low and high) in the cell population, but that both low- and high-CXCR4 expressing cells also expressed PDGFRβ (Figure 2D and E). In addition we performed measurements of Ca^2+^-imaging, stimulating GL15 cells with CXCL12 and then with PDGFBB. Data obtained show that 71.4% of the cells responded with [Ca^2+^] transients to successive applications of CXCL12 and PDGFBB, indicating that a large percentage of GL15 cells functionally responded to CXCR4 and PDGFR activation (traces from 3 representative responsive cells are shown in Figure 2F). To see if this co-expression resulted in synergic activity on cell migration, chemotaxis assays were performed with CXCL12, PDGF or CXCL12 plus PDGF: Figure 2 G shows that no synergism was obtained upon co-treatment, suggesting that CXCL12 and PDGF may act via the same molecular pathways. Figure 2 H shows representative chemotaxis experiment.

### The chemotactic activities of CXCL12 and PDGFBB involve common downstream effectors

The presence of abnormally high levels of active (phosphorylated) forms of PDGFRβ and CXCR4 have been demonstrated in a large number of GBM cell lines and GBM-derived primary cultures [13,16].

**Figure 3:**
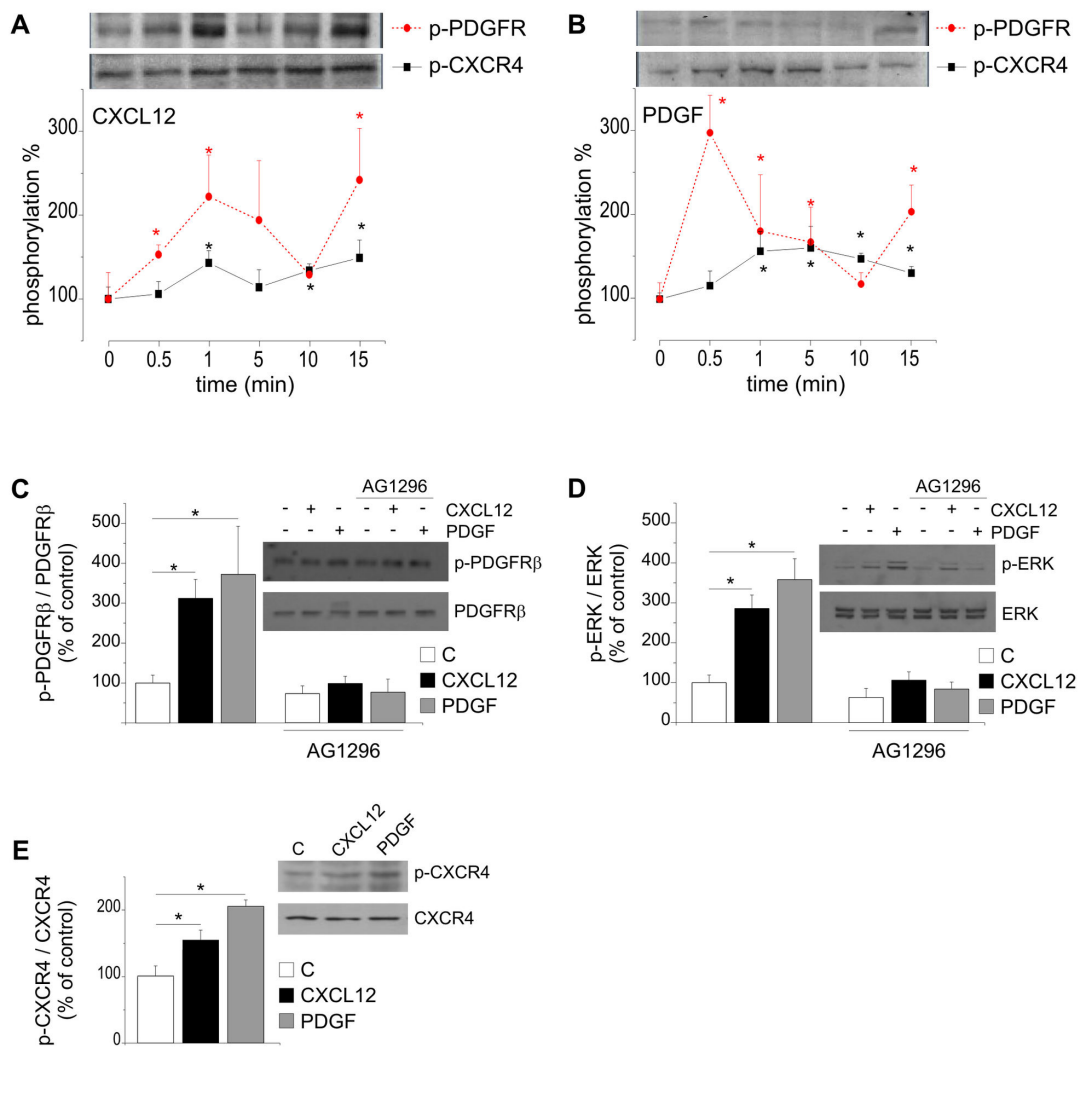
Cross-talk between PDGFRβ and CXCR4. Time course of CXCL12 (50 nM) induced CXCR4 and PDGFRβ phosphorylation (A) and of PDGFBB (50 ng/ml) induced PDGFRβ and CXCR4 phosphorylation (B). Data are the mean of 5 independent experiments. Western blot analyses of CXCL12- (15 min) and PDGFBB- (15 min) induced PDGFRβ (C) and ERK1/2 (D) phosphorylation in GL15 cells in the presence or in the absence of AG1296 (20 µM, 30 min pre-incubation). E) Western blot analysis of CXCL12- (15 min) and PDGFBB- (15 min) induced CXCR4 phosphorylation in GL15 cells. Insets show representative blots. Results are reported as phosphorylated protein/total protein as percentage of untreated cells. Statistical significance: * P<0.05, ** P<0.01, one way Anova or Student’*t* test followed by Mann-Whitney Rank Sum Test for A and B.

To investigate the possibility that the functional cross-talk between PDGFRβ and CXCR4 could be involved in the phosphorylation/activation of these receptors, we analyzed the effect of CXCL12 on PDGFRβ phosphorylation, and of PDGFBB on CXCR4 phosphorylation in GL15 cells. Results shown in Figure 3A demonstrated that CXCL12 (50 nM) efficiently induced Ser 339 CXCR4 phosphorylation (Figure 3A) and also induced a significant increase in PDGFRβ phosphorylation in GL15 cells, with rapid (1 min) and late (15 min) effects. Figure 3 B shows that PDGFBB (50 ng/ml) rapidly activated PDGFRβ phosphorylation and also induced a significant increase in CXCR4 phosphorylation, already detectable at 1 min and maintained up to 15 min. Interestingly, AG1296 efficiently inhibited both PDGFRBB and CXCL12-induced PDGFRβ phosphorylation in GL15 cells (Figure 3C). Also CXCL12- and PDGF-induced ERK1/2 phosphorylation (Figure 3D) was reduced by AG1296. On the other hand, both CXCL12 and PDGFBB -induced (Ser339) CXCR4 phosphorylation was inhibited by AMD3100 (Figure 3 E). These results further confirm a possible cross-talk between CXCR4 and PDGFRβ.

**Figure 4:**
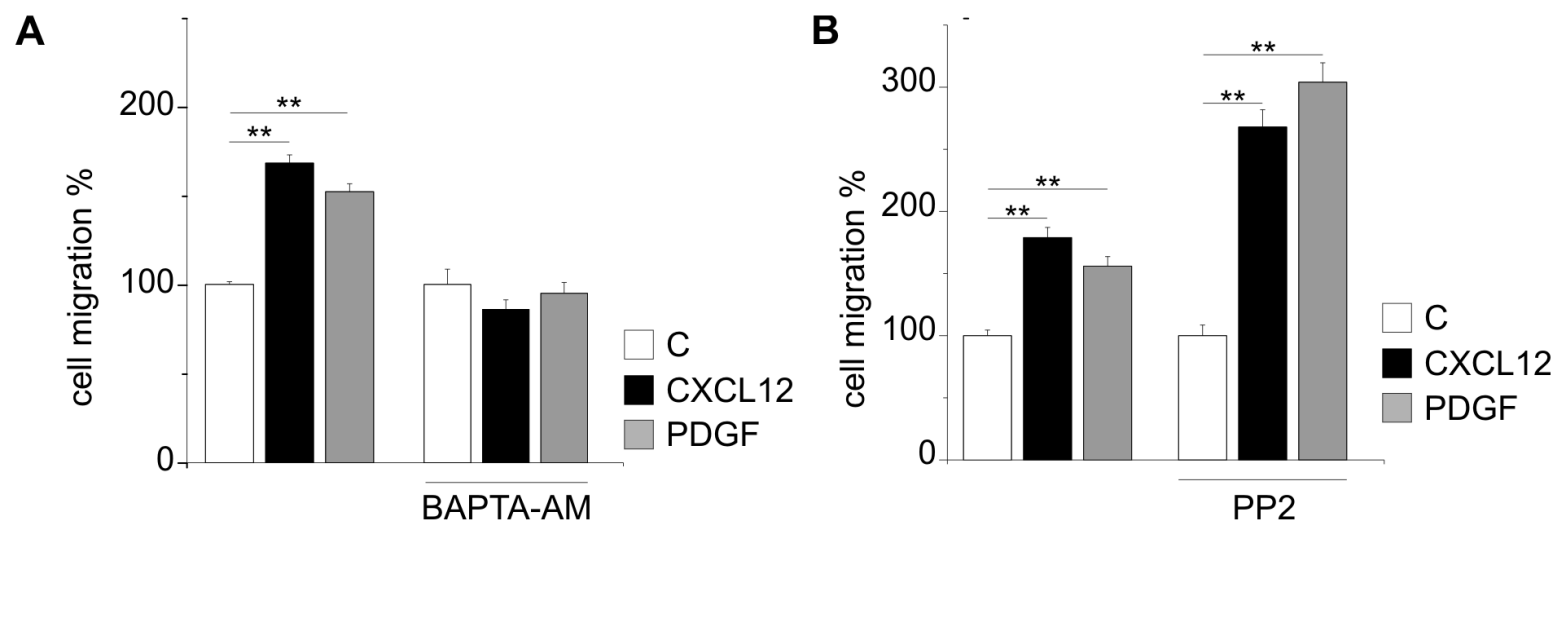
CXCL12 and PDGFBB promote GL15 cell migration through common downstream effectors. Effects of BAPTA-AM (10 µM) (A) and PP2 (2.5 µM) (B), on CXCL12- and PDGFBB-induced GL15 migration. All drugs were preincubated for 15 min. Data are reported as in Figure 1 and are the mean ± S.E. of at least three independent experiments. Statistical significance: *P<0.05, **P<0.01, one way ANOVA.

Trying to identify the common downstream effectors of CXCR4 and PDGFR activation, we analyzed some transduction systems possibly involved in their chemotactic activities. We have previously reported that the Ca^2+^-chelating agent BAPTA abolished the CXCL12-induced, but not the EGF-induced, GBM cell chemotaxis, suggesting that the efficacy of GBM invasiveness might be related to an array of non-overlapping mechanisms activated by different chemotactic agents [25]. To assess whether Ca^2+^ mobilization plays a functional role in the chemotactic response of GL15 cells to PDGF, we performed transwell chemotactic assays in the presence of BAPTA-AM (10 µM). The chemotactic response to both PDGF was completely abolished by the presence of BAPTA-AM (Figure 4A), with no effect on the basal level of cell migration. We further analyzed as possible common pathways src, performing chemotaxis assays with either CXCL12 or PDGF in the presence of PP2 (2.5 µM), an inhibitor of c-src, src-like kinases and c-Abl. Results, reported in Figure 4B, show that PP2, although significantly inhibiting basal migration (n=3, p<0.01), does not affect neither CXCL12- or PDGF-induced chemotaxis, indicating that the crosstalk between CXCR4 and PDGFR is independent of c-src or src-like kinases, as well as from c-Abl activation.

Altogether these data suggest common mechanisms between CXCL12- and PDGF-induced GL15 migration.

### AG1296 inhibits CXCL12-induced chemotaxis in primary cultures from human glioblastoma biopsies

We then investigated whether the cross-talk between CXCR4 and PDGFR and the effects of AG1296 on CXCL12-induced chemotaxis is a general feature of glioblastoma cells or is a phenomenon confined to GL15 glioblastoma cell line. To this aim we performed experiments in freshly resected glioblastoma cells from biopsies. We first analyzed the expression of CXCR4, PDGFRα, PDGFRβ and c-kit by WB. Our results reported in Table 1 show that these proteins are expressed by most of the GBM cells analyzed, although at different levels. Transwell migration assays show that CXCL12 is able to induce significant chemotaxis in two-thirds of the samples, but that this effect is dependent on the activity of PDGFR, since AG1296 is always able to inhibit CXCL12-induced migration (Table 2).

## Discussion

In this paper we demonstrated a functional cross talk between two receptors largely expressed in glioblastomas and playing key roles in tumor cell proliferation, infiltration and angiogenesis, PDGFRβ and CXCR4. We provide evidence of co-expression of CXCR4 and PDGFRβ in a human glioma cell line and in several primary glioblastoma *specimen* from patients. It is well known that the altered expression of growth factors and their receptors and their abnormal activation contributes to glioblastoma aggressiveness acting with autocrine and paracrine mechanisms [13]. PDGF/PDGFR amplification and overexpression is a hallmark of a significant percentage of glioblastoma [31], while mutations leading to constitutive receptor activation are not as common as for other receptors [32]. PDGFRα and β are differently expressed in glioma, and *in silico* survival analysis revealed opposite effects [33] and prognostic alternatives, unmasking the complexity of a therapeutic strategy targeting PDGFRs. It is also known that CXCR4 expression and CXCR4 phosphorylation levels correlate with glioma severity [16]. We have previously demonstrated that glioma migration induced by CXCL12 is specifically abolished by inhibitor of KCa3.1 [25], with mechanisms independent of those activated by EGFR. In this manuscript we provide evidence that CXCL12 signaling involved in chemotaxis of glioma cells is tightly linked to PDGFR signaling since inhibiting CXCR4 activation impairs PDGF-induced chemotaxis and *vice versa*. This effect is specific for PDGFR since EGFR antagonism failed to abolish CXCL12-induced cell migration, even if both EGFR and PDGFR are co-expressed with CXCR4 on the same cells ( [25] and this paper). However, and similarly to what previously described for other cancer cells [30], we observed some functional interaction between EGFR and CXCR4, because AMD3100 abolished EGF induced chemotaxis in GL-15. This result is worth of further future investigation.

We also observed that CXCR4 activation induced the phosphorylation of PDGFRβ and that PDGFBB induced CXCR4 phosphorylation, suggesting receptor co-activation. Examples of cross-activation of PDGFR with GPCRs have been presented in the past for S1PRs [34,35] and P2Y2 [36], with mechanisms only partially defined. Cross activation of tyrosine kinase receptors and GPCRs could imply intracellular or extracellular mechanisms: our evidence of co-expression of PDGFR and CXCR4 on the same cells, as evidenced by FACS analyses and Ca^2+^ imaging experiments, together with the rapid kinetics of receptor cross-activation, would suggest at least possible intracellular pathway rather that the induction of paracrine pathways.

Cross-talk between PDGF and CXCL12 in regulating integrin-dependent pre-B cell proliferation was described [37], suggesting common downstream signaling mechanisms. Our observation that CXCL12- and PDGFBB-induced chemotaxis are both abolished by intracellular Ca^2+^ chelation while not affected by inhibition of c-src, src-like kinases and c-Abl, further suggests common intracellular pathways, strengthening the hypothesis that in glioblastoma CXCR4 and PDGFR are co-activated during single receptor stimulation and cooperate sharing intracellular signaling mechanisms.

## Supporting Information

Figure S1 **Drug specificity.** The specificity of AG1296 was investigated on non transfected (A) or CXCR4 (pCEP)- transfected (B) HEK cells. HEK cells were plated on 35-mm dishes (150.000 cells/dish) and transfected 24 h later using a MagnetofectionTM: Neuromag (OZ Bioscience) procedure, according to manufacturer instruction. Chemotaxis assays were performed 48 h after transfection and lasted 4 h.
The specificity of AMD3100 was investigated performing chemotaxis experiments on two primary GBM cells obtained from patients that were not responsive to CXCL12, GBM12 (C) and GBM13 (D). All indicated inhibitors were pre-incubated for 15 min. Results are reported as percentage of cell migration *vs* control and are the mean ± SE of at least three independent experiments. Statistical significance: *P<0.05, ANOVA one way. CXCL12 (50 nM), PDGFBB (50 ng/ml); AG1296 (20 µM); AMD3100 (1µg/ml).
(TIF)

Figure S2 **Effect of agonists and drugs on serum-starved GBM cells.** Effect of AG1296 (20µM) and AMD3100 (1 µg/ml) on CXCL12- and PDGFBB-induced chemotaxis on serum starved (18 h) GL-15 cells. Results are reported as percentage of cell migration *vs* control and are the mean ± SE of at least three independent experiments. Statistical significance: *P<0.05, ANOVA one way.
(TIF)
