# Supplementary figures and images for: Functional Cross Talk between CXCR4 and PDGFR on Glioblastoma Cells Is Essential for Migration
Source: PLoS One. 2013 Sep 2;8(9):e73426. doi: 10.1371/journal.pone.0073426 (PMC3759384; doi:10.1371/journal.pone.0073426)

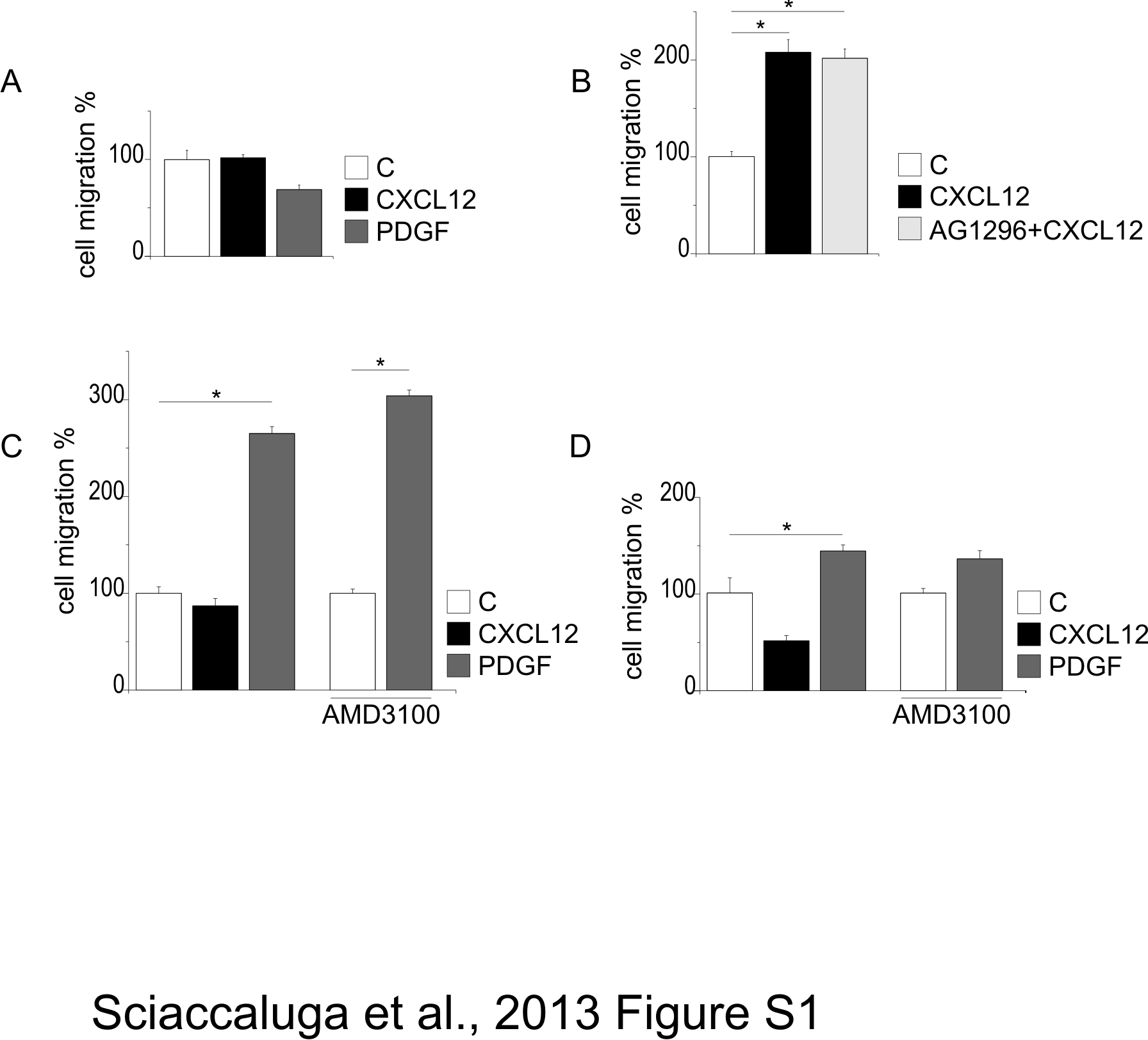

Supplement: Figure S1 — Drug specificity. The specificity of AG1296 was investigated on non transfected (A) or CXCR4 (pCEP)- transfected (B) HEK cells. HEK cells were plated on 35-mm dishes (150.000 cells/dish) and transfected 24 h later using a MagnetofectionTM: Neuromag (OZ Bioscience) procedure, according to manufacturer instruction. Chemotaxis assays were performed 48 h after transfection and lasted 4 h. The specificity of AMD3100 was investigated performing chemotaxis experiments on two primary GBM cells obtained from patients that were not responsive to CXCL12, GBM12 (C) and GBM13 (D). All indicated inhibitors were pre-incubated for 15 min. Results are reported as percentage of cell migration vs control and are the mean ± SE of at least three independent experiments. Statistical significance: *P<0.05, ANOVA one way. CXCL12 (50 nM), PDGFBB (50 ng/ml); AG1296 (20 µM); AMD3100 (1µg/ml). (TIF) [file pone.0073426.s001.tif]

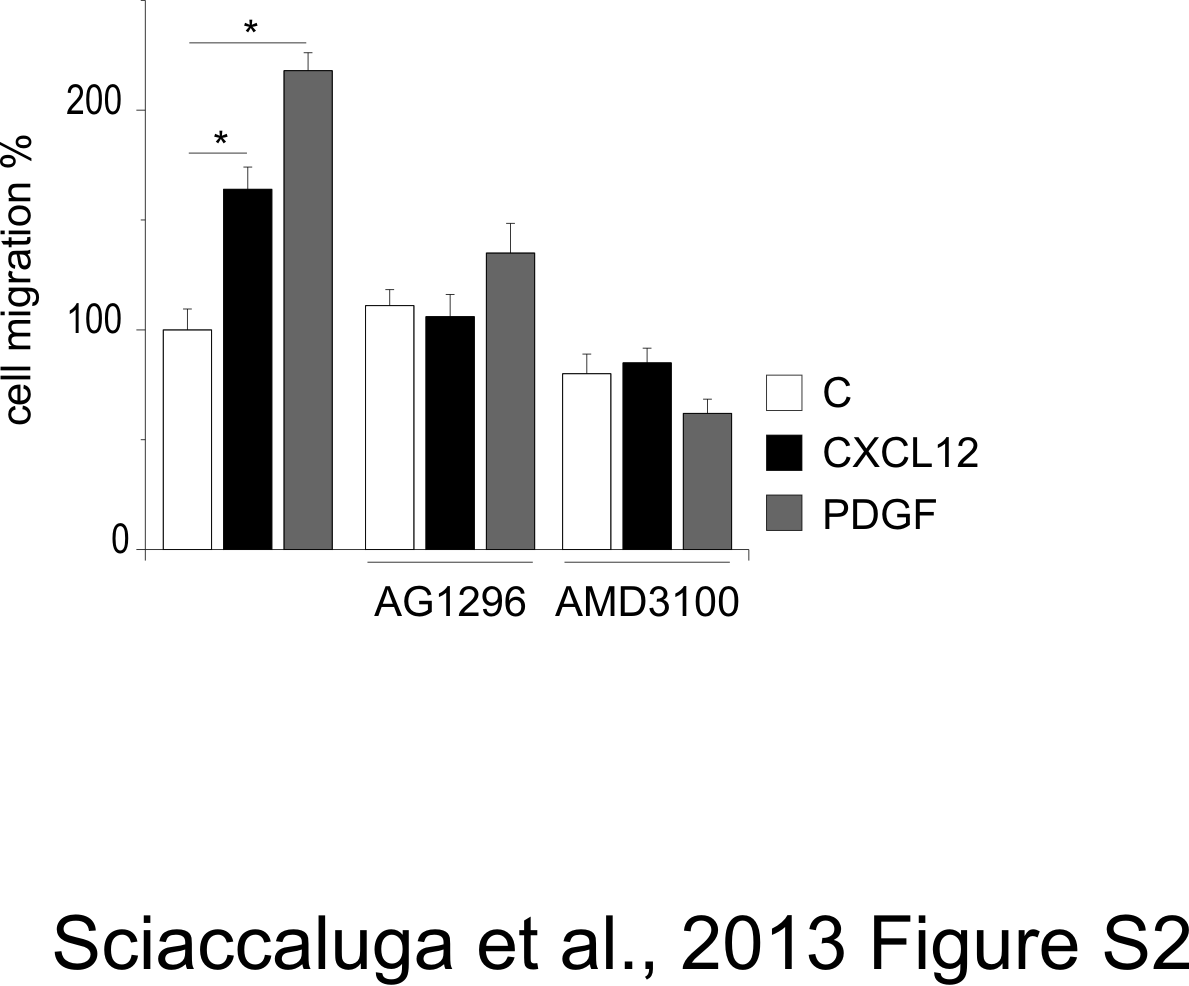

Supplement: Figure S2 — Effect of agonists and drugs on serum-starved GBM cells. Effect of AG1296 (20µM) and AMD3100 (1 µg/ml) on CXCL12- and PDGFBB-induced chemotaxis on serum starved (18 h) GL-15 cells. Results are reported as percentage of cell migration vs control and are the mean ± SE of at least three independent experiments. Statistical significance: *P<0.05, ANOVA one way. (TIF) [file pone.0073426.s002.tif]
